# Emergent anisotropic three-phase order in critically doped superconducting diamond films

**DOI:** 10.1073/pnas.2607730123

**Published:** 2026-05-11

**Authors:** Jyotirmay Dwivedi, Saurav Islam, Jake Morris, Kalana D. Halanayake, Gabriel A. Vázquez-Lizardi, David Snyder, Anthony Richardella, Luke Lyle, Danielle Reifsnyder Hickey, Nazar Delegan, F. Joseph Heremans, David D. Awschalom, Nitin Samarth

**Affiliations:** ^a^ Department of Physics, The Pennsylvania State University, University Park, PA 16802; ^b^ Department of Chemistry, The Pennsylvania State University, University Park, PA 16802; ^c^Electronic Materials and Devices Department, Applied Research Lab, The Pennsylvania State University, University Park, PA 16802; ^d^ Department of Materials Science and Engineering, The Pennsylvania State University, University Park, PA 16802; ^e^Materials Research Institute, The Pennsylvania State University, University Park, PA 16802; ^f^ Q-NEXT, Argonne National Laboratory, Lemont, IL 60439; ^g^ Pritzker School of Molecular Engineering, University of Chicago, Chicago, IL 60637; ^h^ Materials Science Division, Argonne National Laboratory, Lemont, IL 60439; ^i^ Department of Physics, University of Chicago, Chicago, IL 60637

**Keywords:** diamond, superconductivity, magnetotransport, epitaxial films

## Abstract

Two decades since its discovery, superconducting heavily boron-doped diamond (HBDD) still poses fundamental questions that need to be answered to unlock its full potential for quantum applications. We use electrical magnetotransport measurements of critically doped homoepitaxial single crystal HBDD films to reveal signatures of intrinsically granular superconductivity. By studying the dependence of electrical resistivity on temperature and magnetic field vector, we infer that this granularity arises from doping induced disorder. We observe an unexpected three-phase anisotropy in the magnetoresistance, accompanied by a spontaneous transverse voltage (Hall anomaly). Our findings indicate the emergence of an anisotropic order in an otherwise isotropic single crystal HBDD film, offering insights into the mechanism of superconductivity in this quantum material.

## Significance

In heavily boron doped diamond (HBDD), doping induced disorder can lead to inhomogeneity in the superconducting order parameter even in structurally homogeneous samples. We probe this intrinsically granular superconducting state in single crystalline homoepitaxial HBDD films using magnetotransport. Our measurements reveal a three-phase anisotropic order in this disordered superconductor, with distinct symmetries that can be controlled by temperature, magnetic field, and current direction. Understanding the emergence of this anisotropic transport in the superconducting phase of HBDD can lead us toward higher T_c_ in diamond.

Superconducting heavily boron-doped diamond (HBDD) is a promising material for “quantum-on-chip” architectures, with the potential to seamlessly bridge superconducting qubits such as transmons and single spin quantum defects such as nitrogen vacancy (NV) centers (1, 2). Controlling the superconducting behavior of HBDD is important in the context of hybrid quantum technologies (3), thus providing a strong motivation to understand its origins. Boron doping in diamond satisfies Mott’s criterion: $n_{\mathrm{Bc}}a_{H}^{1/3}∼\;0.26$, and its exceptionally small effective Bohr radius ($a_{H}$) in diamond (~0.35 nm) leads to a very high critical concentration $n_{\mathrm{Bc}}∼4\times 10^{20}\;{\text{cm}}^{-3}$ required for the insulator–metal transition, consistent with experimental observations (4, 5). At such high doping levels, both localization and electron correlations become significant, and the transition is sometimes interpreted as an Anderson–Mott transition that proceeds via impurity-band formation (6–9). Below the transition temperature $T_{c}$, HBDD exhibits superconductivity (10–12). Although many characteristics of superconductivity in HBDD are consistent with a weak-coupling BCS superconductor in the dirty limit (13, 14), the influence of electron correlations and disorder near $n_{\mathrm{Bc}}$ remains unresolved. Theoretical work incorporating these effects predicts that $T_{c}\;>\;100\;K$ could be achieved by increasing the concentration of “ordered” boron dopants (15, 16). Although Anderson’s theorem implies that disorder due to nonmagnetic impurities should not affect $T_{c}$, these calculations also show that the increased disorder at higher boron concentrations suppresses $T_{c}$, likely a result of localization and correlations. The maximum $T_{c}$ reported so far in HBDD is only 10.2 K (17), indicating that the role of disorder in the superconducting state of this doped semiconductor remains an open question (5, 13, 18, 19).

In this article, we report the observation of an unexpected magnetotransport anisotropy in [100]-oriented homoepitaxial single crystal HBDD films across the normal metal-superconductor phase transition. Anisotropic transport near the superconducting phase has previously been reported only in nano- and microcrystalline HBDD films, where macroscopic structural inhomogeneities like grain boundaries are the likely source of anisotropy (18, 20, 21). However, in our HBDD samples, structural characterization shows macroscopically homogeneous single crystal growth. Detailed experimental study of electrical transport in these superconducting HBDD films reveals a three-phase anisotropic behavior with distinct symmetries that can be tuned by varying the magnetic field vector (***H***), the direction of the bias current (***J***), and temperature (*T*). We propose that these tunable anisotropic symmetries might be a consequence of emergent “intrinsic” granularity in our critically doped samples, offering a fresh perspective on the impact of disorder in superconducting HBDD (4, 5).

In granular superconductors, the order parameter can vary spatially due to a reduction in pairing amplitude, phase fluctuations, or both (22, 23). This variation can result from two types of inhomogeneity. The first is structural inhomogeneity, seen in granular thin films (24), polycrystalline materials (25, 26), and layered superconductors (27). Here, superconducting grains are physically separated by weak-link grain boundaries, termed “macroscopic” granularity. The second type is electronic inhomogeneity, or “electronic granularity,” where global superconductivity is disrupted by strong electron–electron Coulomb repulsion or strong scattering from doped impurities (28, 29). In these cases, despite a homogeneous crystal structure, doping induced disorder can cause fluctuations in the order parameter creating a network of superconducting islands embedded within a metallic matrix.

## Results

**Fig. 1.**
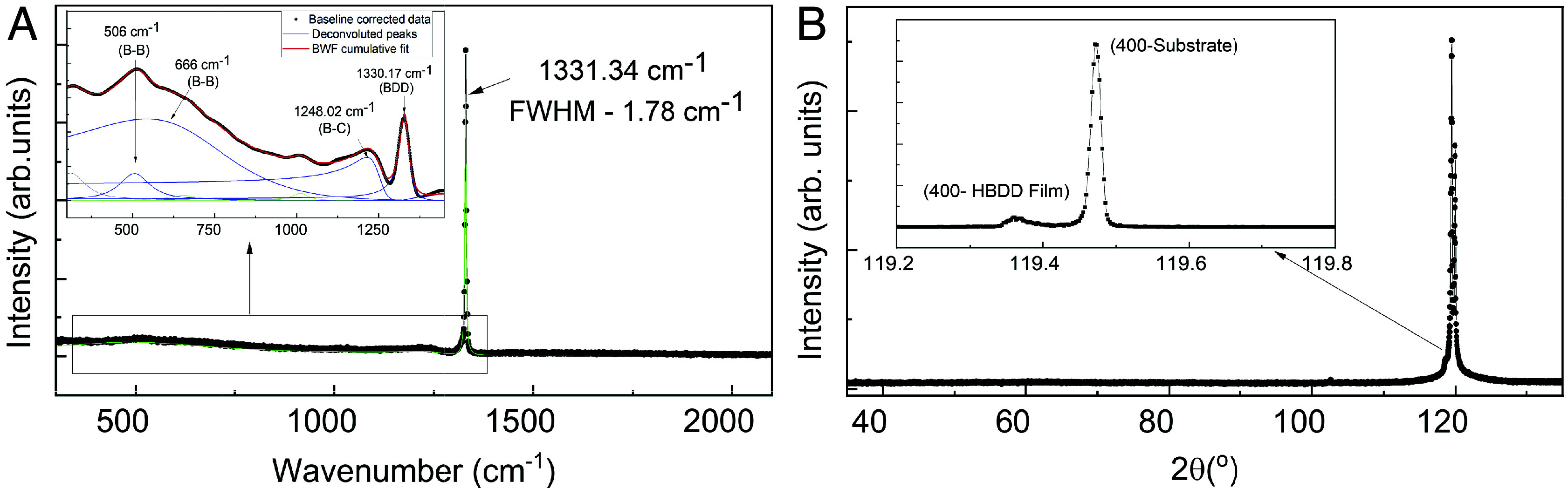
(*A*) Raw Raman spectrum obtained for AE-1 with 532 nm excitation at room temperature shows the sharp diamond zone-center phonon line (ZCP) at 1,331 cm^−1^ a full width half maximum of 1.74 cm^−1^, matching that of the substrate. *Inset* shows the normalized, zoomed-in data with BWF fitting, identifying the modes related to B-doping. (*B*) 2Θ-Θ scan between 35 and 145° using X-ray diffraction shows only peaks consistent with the diamond (400) reflection with no signs of misoriented or polycrystalline growth. *Inset* shows the high-resolution measurements performed on the (400) reflection between 119 and 120°, clearly distinguishing the homoepitaxial boron doped film peak from the substrate at approximately 119.36°.

We synthesize HBDD films on single crystal (100) electronic and optical grade diamond substrates via microwave plasma chemical vapour deposition using BCl_3_ as a dopant precursor gas; this halide growth chemistry contrasts with the more widely used hydrocarbon-based growth achieved using di-borane/tri-methyl borane. A series of samples with thicknesses in the range 0.5 to 20 μm with different boron concentrations are investigated (*Materials and Methods*); here we focus on a 0.5 μm thick film (AE-1) grown on an electronic grade (100) diamond substrate. Fig. 1*A* shows the Raman spectrum measured for AE-1; the inset shows a detailed analysis of this spectrum using the Breit-Wigner-Fano (BWF) function identifying the different Raman modes (*SI Appendix*) (30). The sharp diamond zone-center phonon line (ZCP) at 1,331 cm^−1^ has a full width half maximum of 1.74 cm^−1^, matching that of the substrate which attests to the high quality of our single crystal films. The absence of any G-band signal up to 2,100 cm^−1^ rules out any sp^2^ or amorphous phases of carbon. Previous analysis of Raman spectra of HBDD samples has shown that B is incorporated in the diamond lattice either as a single substitutional B defect or as B–B dimers. Increased doping leads to more complex B incorporation, forming BB-C-BB tetramers and shifting the diamond ZCP to lower wavenumbers (18). An empirical linear fit (31) correlates the B-doping concentration to the Raman peak shift, providing an estimated B concentration of $5\pm 0.4\times 10^{20}\;{\mathrm{cm}}^{-3}$. This positions our sample slightly above the critical concentration ($n_{\mathrm{Bc}}$) required for an insulator to metal transition and for realizing superconductivity. At this critical threshold, doping induced disorder is expected to play a more prominent role, making this sample well-suited to study the competition between disorder and superconductivity in HBDD (4–6, 12, 17, 19, 32).

Fig. 1*B* shows XRD scans performed on a high intensity, monochromated X-ray diffractometer, and the high-resolution data are shown in the inset. The high-resolution measurements clearly distinguish the boron doped film with a larger lattice constant from the underlying (400) substrate peak. A larger lattice constant also shows that the film growth is isotropic, as expected for a (100)-oriented sample (32) and any crystalline anisotropy can be ruled out. Additional TEM analysis on a similar sample also shows diffraction patterns with (100) orientation across a region more than 9 μm wide, further confirming the absence of grain boundaries or other structural inhomogeneity over that length scale (*SI Appendix*, Fig. S2). We also use spatially resolved Raman spectroscopy to confirm uniform boron doping in AE-1 and compare it with other nonuniformly doped samples (*SI Appendix*, Fig. S1) (33).

**Fig. 2.**
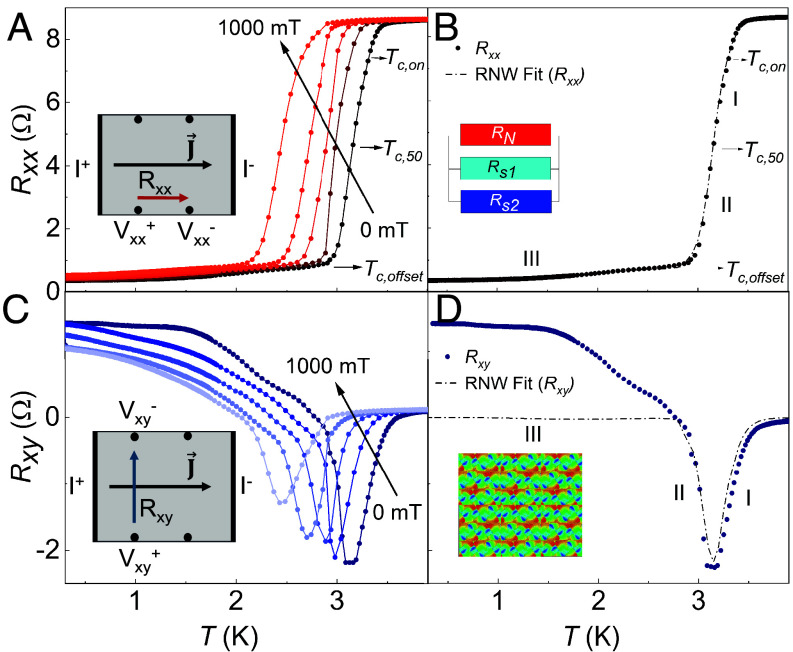
(*A*) Temperature dependence of $R_{\mathrm{xx}}$ at various values of H applied perpendicular to the sample plane with *Inset* showing the contact configuration. The edges of this sample are in the [110] family and the surface is [100] oriented. (*B*) Resistor network model (RNW) fits to $R_{\mathrm{xx}}$ vs. T at 0 mT. (*C*) $R_{\mathrm{xy}}$ vs. T at various values of H applied perpendicular to the sample plane with Inset showing the contact configuration. (*D*) RNW fit for $R_{\mathrm{xy}}$ vs. T at 0 mT using $R_{\mathrm{xy}}$ = $KdR_{\mathrm{xx}}/dT$. A visual representation of intrinsic granularity is shown in the inset of (d) where red and blue regions represent fermionic channels and bosonic islands respectively.

Fig. 2*A* shows the temperature variation of the longitudinal resistance, $R_{\mathrm{xx}}$ at different values of magnetic field, H applied out-of-plane (OP) to the film with the corresponding measurement configuration shown in the inset. The $R_{\mathrm{xx}}\left(T\right)$ data show the onset of superconductivity at $T_{c,\text{onset}}\approx 3.3\;K$ at zero magnetic field. ($T_{c,\text{onset}}$ is defined as the temperature where the $R_{\mathrm{xx}}$ drops to 90% of the normal state value $R_{N}$ at T=4 K.) With increasing H, $T_{c,\text{onset}}$ decreases as expected. However, $R_{\mathrm{xx}}$ does not reduce completely to zero and instead shows a finite resistance of $∼0.1R_{N}$ at $T\leq T_{c,\text{offset}}\approx 2.8\;K$ (Fig. 2*A*). Notably, when the current in our samples is applied orthogonally (in contrast to Fig. 2), $R_{\mathrm{xx}}$ decreases only to $0.8R_{N}$ at $T_{c,\text{offset}}$, rather than reaching $0.1R_{N}$ (*SI Appendix*, Fig. S4). This directional dependence is unexpected because our single-crystal samples should be isotropic. A comparable current-orientation-dependent anisotropic transport in the superconducting phase has been observed in polycrystalline HBDD and was attributed to Bose–Einstein condensation (BEC), supported by density functional theory (DFT) calculations (18). Furthermore, reports of nonzero residual resistance below $T_{c,\text{offset}}$ in polycrystalline HBDD have been interpreted by modeling the superconducting grains, separated by grain boundaries, as a disordered Josephson junction array (26, 34). In such an extrinsically granular superconductor, the temperature variation of $R_{\mathrm{xx}}$ is equivalent to that of a parallel resistor network with competing low resistance bosonic channels and high resistance fermionic channels. We adopt this formalism to fit the temperature dependence of $R_{\mathrm{xx}}$ in our single crystal films. We use one fermionic resistor ($R_{N}$) and two bosonic resistors ($R_{S1}$ and $R_{S2}$) as shown in Fig. 2*B* (*SI Appendix*). Near $T_{c,\text{onset}}$, the fermionic channel dominates (Phase I). Around $T_{c}^{50\%}$ the bosonic channel starts to dominate because it offers lower resistance (Phase II). Finally, below $T_{c,\text{offset}}$, the bosonic channels reach their maximum possible coherence, but residual fermionic channels lead to low nonzero resistance (Phase III).

Since other anisotropic superconductors show anomalous transverse resistance behavior, we also measure the variation of $R_{\mathrm{xy}}$ with temperature. This shows a spontaneous Hall voltage emerging below $T_{c,\text{onset}}$, even at H=0 mT (Fig. 2*C*). This is unexpected because a conventional Hall voltage should appear only above $T_{c,\text{onset}}$ with an OP magnetic field. With decreasing temperature, this spontaneous Hall voltage goes through a minimum at around $T=T_{c}^{50\%}$, where $R_{\mathrm{xx}}=0.5R_{N}$, before reversing sign and then slowly increasing with $T\leq \;T_{c,\text{offset}}$; this phenomenon is known as a “Hall anomaly.” The origins of the Hall anomaly have been debated extensively for other superconductors (35–37), with explanations that include order parameter confinement and vortex flow in high temperature cuprates (38), electronic nematicity in graphene (39), and granularity in Pb films (40). Despite the differences in material systems, if a simplified random Josephson junction array model similar to Fig. 2*B* is used, the Hall anomaly is always proportional to $dR_{\mathrm{xx}}/dT$ with changing temperature (40). This model fits Phases I and II of our $R_{\mathrm{xy}}\left(T\right)$ data very well, as shown in Fig. 2*D* but it deviates significantly in Phase III, suggesting that $R_{\mathrm{xy}}$ may not be completely dependent on $dR_{\mathrm{xx}}/dT$ and a more sophisticated theoretical model is needed. To further confirm the superconducting transition, we used superconducting quantum interference device (SQUID) magnetometry that clearly shows the Meissner effect in our HBDD film in the magnetization vs. magnetic field dependence at T=2.8K (*SI Appendix*, Fig. S3). The variation of M with decreasing temperature also shows the separation of the zero-field cooled (ZFC) and field cooled (FC) magnetic response at around T=2.8K, marking the onset of diamagnetism [See *SI Appendix*, Fig. S3 and (29)].

**Fig. 3.**
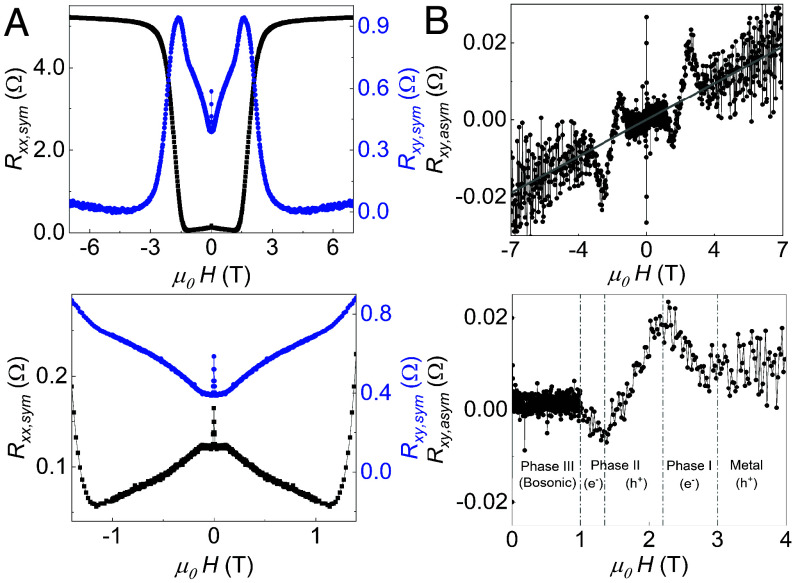
(*A*) The variation of $R_{\mathrm{xx}}$ with applied OP magnetic field at T=2 K (black, left axis). The symmetric component of transverse resistance—$R_{xy,\text{sym}}$ (ETV) (blue, right axis). (*B*) Antisymmetric (Lorentz force) component of $R_{\mathrm{xy}}$ vs. applied field showing hole transport. *Lower* panels show an expanded view of the plots in the *Top* panel. Boson dominated Phase III highlighting the peak feature around zero field (*A*) corresponding to weak localization in residual fermionic channels. *Lower* panel of (*B*) shows different Hall coefficients with different transport phases, marked as metal, I, II, and III where the carrier type switches from electron to hole to electron across the three phases of granular superconducting transition with reducing free carrier concentration. This suggests that the fermionic channels “shrink” as bosonic channels “expand” leading to lower resistance.

The three-phase granularity is further reinforced by the longitudinal magnetoresistance (MR) [$R_{\mathrm{xx}}$ (H)] and transverse MR [$R_{\mathrm{xy}}$ (H)] data (Fig. 3). $R_{\mathrm{xx}}$ increases with increasing magnetic field when the OP H exceeds $H_{c1}$, with normal state resistance restored above $\mu_{0}H_{c2}\approx 3\;T$ (Phase I). In the superconducting state ($0\leq H\leq H_{c1}$), the residual $R_{\mathrm{xx}}$ decreases with increasing field at different rates in Phases II and III, with a sharp peak around zero field (Fig. 3*A*, black curve). The peak near H=0 mT and negative low-field MR below $H_{c1}$ is a signature of weak localization that attests to the coexistence of disorder in the superconducting state of our samples (4, 5, 13, 21, 28). Note that in the normal state, $R_{\mathrm{xy}}$ shows a conventional “antisymmetric” Hall resistance with respect to the OP magnetic field (*SI Appendix*) (12). Within the superconducting transition, $R_{\mathrm{xy}}$ becomes symmetric with respect to the OP magnetic field due to the Hall anomaly, manifesting as an “even-in-field transverse voltage” (ETV) (40–43). We separate the conventional “antisymmetric” component of $R_{\mathrm{xy}}$ as $R_{xy,\text{asym}}$ and anomalous “symmetric” component of $R_{\mathrm{xy}}$ as $R_{xy,\text{sym}}$ (29). $R_{\mathrm{xx}}$ is also symmetrized into $R_{xx,\text{sym}}$ to remove any $R_{\mathrm{xy}}$ contribution due to contact misalignment. The ETV (Fig. 3*A*, blue curve) is found to be an order of magnitude larger than the conventional Hall effect. It also exhibits a peak near zero field (Fig. 3*A*, *Lower* panel), mirroring the behavior of $R_{\mathrm{xx}}$ and supporting the presence of weak localization (5, 13, 19, 21, 25, 28).

**Fig. 4.**
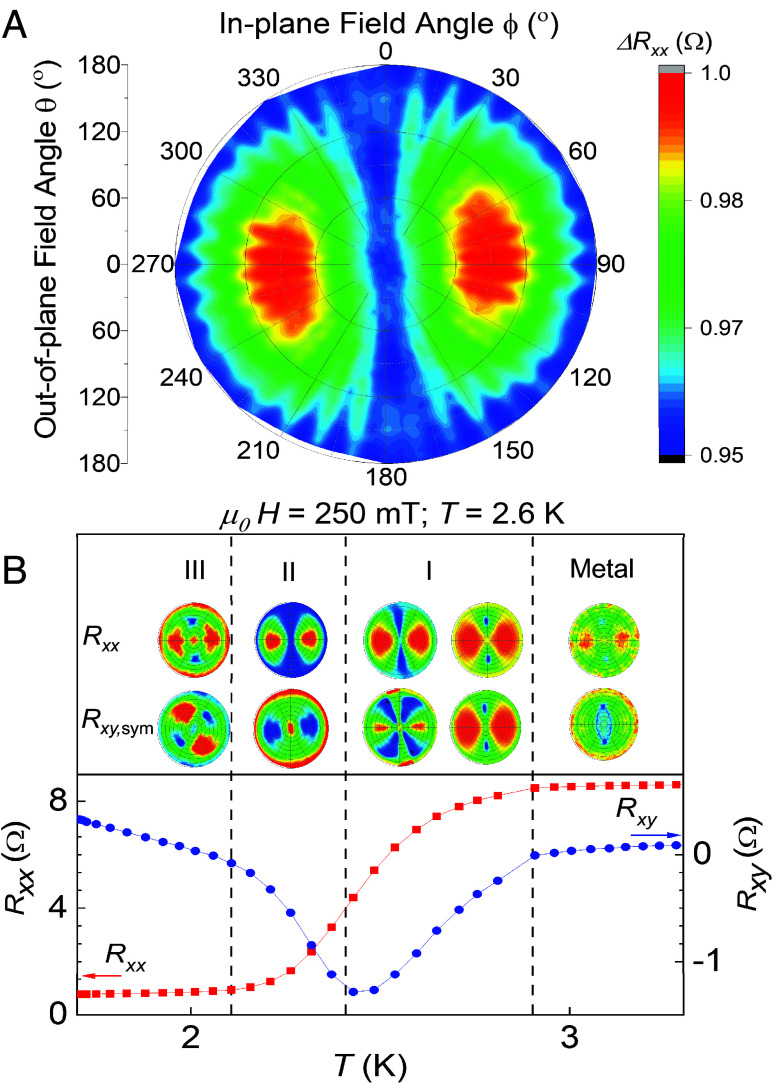
Angular MR measurements. (*A*) Azimuthal equidistant projection illustrates the angular anisotropy in $R_{xx,\text{sym}}$ as the magnetic field ***H*** is rotated from (out-of-plane) OP to (in-plane) IP through angle θ at T=2.62 K and |H|=250 mT. The projection center represents resistance for OP field in the +z direction (North pole), while its circumference corresponds to OP field in the -z direction (South pole). The middle ring (equator) denotes resistance for purely IP field in the sample plane. The vertical (0 to 180°) and horizontal (90 to 270°) diameters map field rotation paths parallel and perpendicular to the current, respectively. Regions of high resistance (red) appear in a 45° conical zone near the equator, indicating dominant IP field components, whereas low resistance (blue) is seen near the poles, indicating dominant OP field components. (*B*) Evolution of anisotropy symmetry and magnitude in the MR across different resistive phases demarcated by dotted lines. The first row in the *Top* panel shows the longitudinal resistance $R_{\mathrm{xx}}$ and the second row shows the symmetric transverse resistance $R_{xy,\text{sym}}$ or the Hall anomaly. The polar plots shown here have the same color intensity scale as used in (*A*).

Different carrier properties extracted from Fig. 3*B* are summarized in *SI Appendix*, Table S2. The Ioffe-Regel parameter ($k_{F}l_{e}$), is commonly used to quantify disorder where $k_{F}l_{e}<1$ indicates localization and strong disorder. Although, it may not be an accurate metric in case of doped superconductors. In our sample, $k_{F}l_{e}$ increases from 2.39 in the normal state to 11.7 in the superconducting state, indicating that the localization weakens across the superconducting transition. Previous reports have suggested that weak localization might act as a precursor to superconductivity in nanocrystalline HBDD (44, 45). The coexistence of localization and superconductivity has been widely investigated in two-dimensional disordered superconductors, revealing unconventional phenomena such as Cooper-pair localization, pseudogap phases, and multifractal superconductivity (28, 46, 47). These findings motivated us to further explore the emergent granular superconductivity in our samples by measuring the MR at fixed temperatures while rotating the magnetic field vector over the unit sphere in 5° increments. We plot these data as an azimuthal equidistant projection as shown in Fig. 4*A*. (This is similar to the projection of a 3D globe onto the 2D map of Earth as seen in the United Nations emblem.)

For purely 3D isotropic superconductors, no change is expected in resistance as the magnetic field angle is changed. For 2D superconductors, an OP-to-IP field rotation results in a decrease in resistance because electron pairing is more robust against magnetic field in the IP direction (48). Here, we see the *opposite* effect where OP-to-IP field rotation increases resistance when ***H*** ⊥ ***J***. A similar observation was reported in 3D polycrystalline HBDD samples that have vertical grain boundaries (20). However, our HBDD film is a 0.5 μm thick 3D single crystal with no evident grain boundary formation indicating that the anomalous MR anisotropy in HBDD emerges due to intrinsic granularity because our samples are at critical doping concentration ($n_{\mathrm{Bc}}$). Similar anisotropy maps, albeit with different symmetries, were obtained for other samples with nonuniform boron concentration (*SI Appendix*, Fig. S10). Tracking the evolution of this anisotropic angular MR behavior at different ***H - T*** points can provide further insight into the nature of the three-phase superconducting transition identified in Fig. 2 *B* and *D*. We plot the equidistant azimuthal projections of the angular MR at T = 300 mK, 2.1 K, 2.6 K, 2.9 K, and 4 K with different magnitude of magnetic field ($\begin{array}{c} \mu_{0}H=250\;\mathrm{mT},\;500\;\mathrm{mT},\; \end{array}$ $\begin{array}{c} 750\;\mathrm{mT},\;\mathrm{and}\;1,000\;\mathrm{mT} \end{array}$). Then, we extract $R_{xx,\text{sym}}$, $R_{xy,\text{asym}}$, and $R_{xy,\text{sym}}$ from each, resulting in a dataset of more than 50 plots (*SI Appendix*, Figs. S6–S8). Careful inspection indicates that the angle-dependent MR anisotropy shows similar symmetry and magnitude for all ***H - T*** points lying within a single phase. The anisotropy varies as we move across the three phases. This is visualized in Fig. 4*B* where the first and second rows in the top panel represents $R_{xx,\text{sym}}$, and $R_{xy,\text{sym}}$, respectively. The reference blue and red curves are extracted from Fig. 2*A* and represent the variation of $R_{\mathrm{xx}}$ and $R_{\mathrm{xy}}$ with temperature for $\mu_{0}H_{\mathrm{OP}}=1,000\;\mathrm{mT}$.

In the normal metallic state at 4 K, the resistance is nearly angle-independent, indicating that the observed three-phase anisotropy only emerges below $T_{c,\text{onset}}$. In Phase I ($T_{c,\text{onset}}=2.9\;K$), both $R_{xx,\text{sym}}$ and $R_{xy,\text{sym}}$ exhibit twofold symmetry, with high resistance lobes when ***H_xy_*** ⊥ ***J*** and low resistance pockets for ***H_xy_***∥ *J*; the large angular MR here points to predominantly fermionic transport. Near the Phase I/II boundary (T=2.62 K), $R_{\mathrm{xx}}$ falls below $0.5R_{N}$ and $R_{\mathrm{xy}}$ reaches a minimum, marking the emergence of bosonic transport channels that expand the low-resistance regions in $R_{xx,\text{sym}}$ and produce a distinct four-lobe symmetry in $R_{xy,\text{sym}}$. Deeper into Phase II (2.1 K), bosonic channels dominate, further enlarging low-resistance areas in $R_{xx,\text{sym}}$ while maintaining twofold symmetry, whereas $R_{xy,\text{sym}}$ shows complementary color alignment: blue lobes for ***H_xy_*** ⊥ ***J*** and red for ***H_xy_*** ∥ ***J***. At 300 mK in Phase III, bosonic transport dominates, lowering angular MR contrast in both $R_{xx,\text{sym}}$ and $R_{xy,\text{sym}}$; twofold symmetry persists in $R_{xx,\text{sym}}$ while $R_{xy,\text{sym}}$ displays a 45° rotation about the polar axis.

## Discussion

The observation of finite $R_{xx}\;below\;T_{c,offset}$ and the presence of a Hall anomaly indicate that superconductivity is inhomogeneous in our samples. The resistor network models help us identify a three-step transition from metallic to inhomogeneous superconducting phase as shown by regions I, II, and III in Fig. 2 *B* and *D*. These models, developed for extrinsically granular superconductors with structural inhomogeneity, might suggest that the inhomogeneity in our samples stems from statistical variation in boron concentration. However, structural characterization using TEM shows that these samples have uniform doping over length scales of greater than 9 μm without grain boundaries. This is further supported by XRD measurements that only show a (100) phase. It is more likely that the doping concentration in our sample ($5\;\pm \;0.4\;\times \;10^{20}\;cm^{-3}$) places it just above the critical point ($n_{\mathrm{Bc}}∼4\times 10^{20}\;{\mathrm{cm}}^{-3}$) of the insulator–metal transition where doping induced disorder and electron–phonon coupling compete with each other, giving rise to an “intrinsically” granular superconducting phase (4, 5, 19). This competition manifests as different phases of negative MR variation shown in Fig. 3 that points to the coexistence of weak localization and superconductivity in our sample.

The angular MR evolution across the superconducting transition phases gives further insight into this competition between disorder and superconductivity, demonstrating tunable superconducting granularity in homoepitaxial HBDD films. We observe these anisotropies in different samples with varying symmetries and boron distribution, revealing an emergent electronic order dependent on the relative orientation of current and magnetic field, as well as temperature (*SI Appendix*, Figs. S9 and S10). Prior studies of nano- and polycrystalline HBDD thin films, where the structural granularity dominates, have reported some form of anisotropy in the superconducting phase (4, 18, 20, 45). The observed anisotropy was weaker in those HBDD samples, possibly due to boron concentration exceeding 10^21^ cm^−3^ and thus a stronger electron–phonon coupling as most of the impurity states are fully delocalized, hence masking any contribution from disorder induced localization (4, 5, 17, 49). By studying samples in the critical doping regime and also eliminating structural granularity, we isolate electronic granularity, revealing three tunable phases with distinct symmetries of an intrinsic electronic order in this disordered system. Fig. 2 *D* (*Inset*) visualizes this as a network of superconducting puddles (blue), fermionic regions (red), and incoherent preformed Cooper pairs (green). Without structural pinning, temperature and electromagnetic fields can tune the size and shape of these channels, leading to the three observed phase symmetries (Fig. 4).

Our observation of strongly anisotropic transport in intrinsically granular and critically doped superconducting homoepitaxial HBDD films highlights the role of disorder in the superconducting phase of samples near the insulator–metal transition, clearly indicating that boron doping in diamond induces fluctuations and inhomogeneity in the superconducting phase, even in highly ordered, crystalline HBDD. The anisotropic order emerging within this electronic disorder can be tuned by temperature and magnetic field. Probing this anisotropy using techniques other than transport, such as scanning tunneling microscopy, is crucial to gain a deeper understanding of how disorder affects granular superconductivity in HBDD, especially in the context of multifractal superconductivity and pseudogap states (46, 47). This might also provide a route to higher critical temperatures, as suggested by theoretical work on doping-induced disorder in superconducting HBDD (15). Additionally, the results shown here may be relevant in the broader context of superconductivity in other doped group IV semiconductors (50, 51). Finally, a complete understanding of the interplay between electron correlations, dopant-induced disorder, and the accompanying superconductivity in HBDD is relevant for applications in quantum technologies. For example, the recent demonstration of uniformly distributed NV centers in the vicinity of a HBDD layer (52) allows us to envision exploiting “intrinsically” inhomogeneous superconductivity to facilitate information transfer across NV center qubits by using a magnetic field to control whether the NVs interact with fermionic or bosonic patches.

## Materials and Methods

HBDD films were deposited on single-crystal (100) (±3° miscut) electronic-grade diamond substrates (10^14^ cm^−3^ defect density) and optical-grade substrates (scratch-resistant, low-birefringence) in a microwave plasma chemical vapour deposition chamber. A JRC Model NJA2103A microwave generator produced an ellipsoidal plasma at 1.4 to 1.6 kW power on top of the molybdenum susceptor holding the substrate. An infra-red thermometer measured the substrate temperature. Substrates were cleaned with acetone and isopropanol prior to growth, followed by an in-situ 95 sccm hydrogen plasma surface etch for 10 min at 900 °C. Once the growth temperature (1,200 °C) was achieved, 5 sccm CH_4_ and 20 to 75 sccm BCl_3_-Ar (1,000 ppm) were introduced into the chamber for varying growth durations (25 to 60 min), resulting in sample thicknesses of 0.5 to 20 μm as measured by a micrometer gauge. A summary of all samples grown is provided in *SI Appendix*, Table S3. The main manuscript study focuses on a 0.5 μm thick sample labeled AE-1 grown on an electronic grade substrate. Other samples—E-15 and E-16—discussed in *SI Appendix* were grown on optical grade substrates.

Electrical resistivity measurements were performed using lock-in amplifiers operating at 17.77 Hz in an Oxford Triton-He3 cryostat equipped with a vector magnet for rotating magnetic fields. Magnetoresistance (MR) characteristics were measured in a Quantum Design DynaCool Physical Property Measurement System using a digital lock-in scheme. Magnetic susceptibility measurements were conducted in a Quantum Design Magnetic Property Measurement System in the OP magnetic field configuration. Raman measurements were performed at room temperature with 532 nm laser excitation in a Horiba LabRAM setup.

## Supplementary Material

Appendix 01 (PDF)

## Data Availability

Experimental data have been deposited in scholarsphere.psu.edu (53). All other data are included in the manuscript and/or *SI Appendix*.
