# Supplementary material for: Emergent anisotropic three-phase order in critically doped superconducting diamond films
Source: Proc Natl Acad Sci U S A. 2026 May 11;123(20):e2607730123. doi: 10.1073/pnas.2607730123 (PMC13187776; doi:10.1073/pnas.2607730123)
Supplement: Supplementary file 1 — Appendix 01 (PDF) [file pnas.2607730123.sapp.pdf]

PNAS

Supporting Information for

Emergent anisotropic three-phase order in critically doped superconducting diamond films

Jyotirmay Dwivedi, Saurav Islam, Jake Morris, Kalana D. Halanayake, Gabriel A. Vázquez-Lizardi, David Snyder, Anthony Richardella, Luke Lyle, Danielle Reifsnyder Hickey, Nazar Delegan, F. Joseph Heremans, David D. Awschalom, Nitin Samarth

Nitin Samarth, David Awschalom
E-mail: nxs16@psu.edu, awsch@uchicago.edu

This PDF file includes:

Supporting text
Figs. S1 to S10
Tables S1 to S3
SI References

Supporting Information Text

Raman Spectroscopy

Raman measurements were carried out in a confocal Horiba LabRAM setup (100× magnification) with 532 nm excitation at room temperature. In Fig. S1(a), the raw Raman spectrum from AE-1 is shown. As the spectrum is dominated by the substrate's diamond signal, this baseline was subtracted, and the data were normalized for clarity. The normalized Raman spectrum in Fig. 1*A* (inset) was fitted using the Breit-Wigner-Fano (BWF) function (1):

$$y = y_0 + \frac{H\left(1 + \dfrac{x - x_c}{q\omega}\right)^2}{\left(1 + \dfrac{x - x_c}{\omega}\right)}. \quad [1]$$

Here, y_0 is the baseline, H is the peak height, x_c is the peak center, and ω is the full-width half-maximum (FWHM). The parameter q is the Fano symmetry factor, where $|q| \propto 1/\text{asymmetry}$. In Fig. 1*A* (inset), the spectrum around 505 cm^{-1} and 660 cm^{-1} shows the B vibrational modes, while the asymmetric peak at 1248 cm^{-1} with a low value of $q = -1.33$ corresponds to the B–C phonon density of states. The diamond zone-center phonon line (ZCP) at 1330 cm^{-1} is shifted from its usual value of 1332 cm^{-1} with $q = -7.48$, a consequence of phonon softening due to heavy B doping. Deconvoluted peaks from the fitted curve are displayed in blue to highlight the Fano asymmetry. Table S1 summarizes the peak parameters (in cm^{-1}) used for fitting (the Fano asymmetry factor is dimensionless).

Structural Analysis

Sample crystallinity was analyzed using transmission electron microscopy (TEM) and selected-area electron diffraction (SAED) as shown in Fig. S2. An FEI Scios 2 dual-beam Ga^+ focused ion beam–scanning electron microscope (FIB-SEM) was used to create electron-transparent lamellae for TEM and SAED analysis. The lamellae were extracted from regions patterned for electrical transport measurements and subsequently thinned to electron transparency using FIB accelerating voltages of 30 kV and 5 kV, with a final cleaning step at 2 kV. TEM and SAED measurements were performed on an FEI Talos F200X at an accelerating voltage of 200 kV. Using a selected-area aperture, SAED patterns were collected from 4 µm regions of the sample.

Further, a $2\Theta - \Theta$ scan was measured between 35.0-145.0° with a step size of 0.026° on a 240.00 mm radius Malvern Panalytical Empyrean X-ray diffractometer equipped with a line source [Cu K-α 1-2 (1.5405980/ 1.5444260Å)] X-ray tube at 45.0 kV and 40.0 mA. The incident beam path included an iCore BBHD® optic with 0.0300 radian Soller slits and a 0.25° divergence slit. The diffracted beam path incorporated dCore® optics with 0.0400 radian Soller slits and a 0.25° anti-scatter slit. A PIXcel3D-Medipix3 1x1 detector operating in scanning line (1D) mode was used. PHD lower and upper levels were set at 4.02 and 11.27 keV respectively. The measurement shows only peaks consistent with the diamond (400) reflection with no signs of misoriented or polycrystalline growth as shown in Fig. 1*B*. Further high resolution measurements were performed on the (400) reflection between 119.0-120.0° 2Theta-Omega with a step size of 0.002° on a 320.00 mm radius Malvern Panalytical X'Pert3 MRD four circle X-ray diffractometer equipped with a point source [Cu K-α 1-2 (1.5405980/ 1.5444260Å)] X-ray tube at 45.0 kV and 40.0 mA and an incident 4xGe220 Cu symmetric monochromator with cross-slits set to 1 mm x 1 mm. A PIXcel3D 1x1 detector operating in receiving slit mode was used with an active length of 0.495 mm. PHD lower and upper levels were set at 4.02 and 11.27 keV respectively. This measurement clearly distinguishes the homo-epitaxial boron doped film peak from the substrate at approximately 119.36°.

Transport and Magnetization

Conductance of doped semiconductors near the metal-insulator transition can be characterized by the following function (2):

$$\sigma = \sigma_0 + A\sqrt{T} + BT$$

Here, σ_0 is the 0 K conductance of a disordered metal, $A\sqrt{T}$ accounts for Coulomb repulsion, and BT dependence arises from quantum corrections dominated by electron-electron interactions. We fitted our resistance versus temperature data from 5 K to 300 K using this function in Fig. S3(a). The finite value of B indicates that electron-electron interactions play an important role in the temperature dependence of the metallic state in our samples (3, 4).

Magnetic moment versus temperature (and magnetic field) data recorded for the sample are labeled as Raw Data. A bare substrate was then loaded onto the same holder, and the measurement was repeated to obtain the Substrate+Holder contribution. This contribution was subtracted directly from the Raw Data to yield the corrected data. In Fig. S3(b), data were recorded with a 3 mT out-of-plane field for the field-cooled cycle (red curve). All data in Fig. S3(c) were recorded at 2 K.

Resistor Network Models

For fitting the R_{xx} vs T data shown in Fig. 2*B*, we use the phenomenological resistor network model used by Zhang *et al.*(5). Metallic state resistance (R_N) as a function of temperature is modeled by:

$$R_N = r_n \left[-2 \int_{\Delta T}^{\infty} g(\epsilon) f'(\epsilon)\, d\epsilon \right]$$

$$\Delta(T) = \Delta_0(0)\left(1 - \frac{T}{T_c^{onset}}\right)^{0.5}$$

$$g(\epsilon) = \left[\frac{E}{E^2 - \Delta T^2}\right]; f'(\epsilon) = -\frac{1}{k_B T}\left[\frac{exp(\frac{\epsilon-\mu}{k_B T})}{(exp(\frac{\epsilon-\mu}{k_B T})+1)^2}\right]$$

Here, $\Delta(T)$ corresponds to the superconducting energy gap with $\Delta_0(0)$ being the gap at $T = 0$ K, $g(\epsilon)$ is the density of states of a single fermion, and $f'(\epsilon)$ is the derivative of the Fermi-Dirac distribution. The bosonic channels can be empirically described as:

$$R_s = r_m + r_s\ exp\left(\frac{T}{T_{cg}} - 1\right)^{\eta}$$

Here, r_m is the residual resistance due to fermionic channels, r_s is a constant, T_{cg} is the temperature at which global coherence is achieved, and η is the exponent determining the sharpness of the transition. We fit our resistance versus temperature data by considering three such resistors operating in parallel—one fermionic resistor R_N and two bosonic resistors R_{s1} and R_{s2}, as shown in Fig. 2*B*. This combination is deduced from the fact that our resistance versus temperature curve shows two transitions: a sharp transition at around 3.3 K and a shallower transition at around 2.9 K, marking the onset of Phase III. We fix the values of $T_c^{\text{onset}} = 3.3$ K, $T_{cg1} = 1.8$ K, $T_{cg2} = 0.59$ K, and $r_n = 8.7\ \Omega$. Our fitting parameters yield: $r_{m1} = 0.92\ \Omega$, $r_{m2} = 0.633\ \Omega$, $r_{s1} = 1171\ \Omega$, $r_{s2} = 0.073\ \Omega$, $\eta_1 = 17.4$, and $\eta_2 = 3.22$. The zero-temperature superconducting gap $\Delta_0(0)$ is 0.8 meV. These parameters are comparable to fits reported in the literature for polycrystalline samples using the same model (5–7), indicating that our single-crystal films exhibit doping disorder-mediated granular superconductivity.

Similarly, we use a resistor network model that accounts for inhomogeneous superconductivity leading to the Hall anomaly (8):

$$R_{xy} = \Delta T \frac{\partial R_{xx}(T,H)}{\partial T} + \Delta H \frac{\partial R_{xx}(T,H)}{\partial H}$$

Here, ΔT and ΔH are constants accounting for the difference in T_c and H_c of different superconducting regions. For H = 0, the relation reduces to :

$$R_{xy} = K \frac{dR_{xx}(T,0)}{dT}$$

This formula is used to fit the R_{xy} variation with temperature in Fig. 2*D*.

Magnetoresistance

We take the ratio of R_{xy}/R_{xx} at 4 K with zero magnetic field and obtain a factor of 0.188 to account for contact misalignment (9):

$$R_{xy} = R_{xy} - 0.188 R_{xx}$$

We then symmetrize the magnetoresistance data using the formula:

$$R_{xx,sym}(\boldsymbol{H}) = (R_{xx}(\boldsymbol{H}) + R_{xx}(-\boldsymbol{H}))/2$$

Similarly, for the transverse magnetoresistance R_{xy}, the low-magnitude antisymmetric component $R_{xy,\text{asym}}$ corresponding to the Lorentz force effect on charge carriers is calculated by:

$$R_{xy,asym}(\boldsymbol{H}) = (R_{xy}(\boldsymbol{H}) - R_{xy}(-\boldsymbol{H}))/2$$

and the dominant symmetric (ETV) component— $R_{xy,\text{sym}}$ is calculated by:

$$R_{xy,sym}(\boldsymbol{H}) = (R_{xy}(\boldsymbol{H}) + R_{xy}(-\boldsymbol{H}))/2$$

These corrections also account for inevitable sample tilt.

A summary of fermionic channel properties extracted from Fig. 3*B* is provided in Table S2. Here, ζ is the coherence length extracted from the H–T phase diagram (Fig. S5).

Transport in the Orthogonal Current Direction

Noting that the Hall anomaly has primarily been observed in superconductors that explicitly possess anisotropy, we searched for in-plane anisotropy in our HBDD films by performing resistance measurements with current flowing in the orthogonal direction ($\boldsymbol{J} \parallel$ (110)) compared to the original configuration ($\boldsymbol{J} \parallel$ (1-10)). The data in Fig. S4 reveal highly anisotropic behavior: R_{xx} shows the onset of a superconducting transition but fails to reach a zero-resistance state, saturating instead at around $0.8R_N$. The Hall anomaly persists in this configuration, albeit with reduced magnitude. Crucially, no anisotropy exists in the normal state—R_N is similar in value ($\sim 7\ \Omega$) for both current directions, with anisotropy emerging only upon the onset of superconductivity ($T \leq T_{c,\text{onset}}$). As diamond is an isotropic cubic crystal, this direction-dependent transport likely originates from disorder-induced localization. We also constructed a phase diagram for the ($\boldsymbol{J} \parallel$ (110)) configuration, analogous to Fig. 4*B* for $\boldsymbol{J} \parallel$ (1-10). Fig. S4(a) shows the same three-phase behavior but with more pronounced 4-lobed symmetries compared to the $\boldsymbol{J} \parallel$ (1-10) case.

WHH Fit

If HBDD is considered a weakly coupled type-II BCS superconductor, a Werthamer-Helfand-Hohenberg (WHH) fit can quantify the "anisotropic robustness" of bosonic channels by calculating the upper critical field for both current directions using the H–T phase diagram in Fig. S5 (10). Here, $T_{c,\text{inflection}}$ is defined as the temperature where the Hall anomaly (R_{xy}) changes slope at the Phase I–II boundary. We calculate the upper critical field for both current directions using the BCS-WHH estimate, where the reduced temperature exponent $(1-(T/T_c))$ is constant. With the Maki parameter $\alpha = 0$ and $\lambda_{so} = 0$ (neglecting Pauli paramagnetism and spin-orbit coupling), we find $H_{c2}(010) > H_{c2}(110)$. This is counterintuitive, as the more resistive direction is expected to exhibit more fluctuations and thus a lower critical field. Setting $\alpha = 0.65$ for the (1-10) H–T phase diagram aligns it with the (110) direction's H–T curve, suggesting that Pauli paramagnetism and spin-orbit coupling may influence the more resistive direction.

We also considered a scenario where the reduced temperature exponent ω is a fitting parameter. The (1-10) [RT-A] direction now exhibits a higher critical field than the (110) direction, as expected, with $\omega = 1.57$—close to the BCS value of 1.5. In contrast, $\omega = 2.56$ for the (110) direction deviates significantly from the BCS prediction. Finally, we use the formula:

$$\xi(0) = \sqrt{\frac{\Phi_0}{2\pi H_{c2}(0)}}$$

to calculate the coherence length in Table 1, where $\xi(0)$ is the coherence length, Φ_0 is the magnetic flux quantum, and H_{c2} is the critical field extracted from the WHH fit.

Polar Plot Dataset

Figures S6–S8 show the representative dataset of magnetoresistance recorded at different magnetic fields and temperatures for one sample. This dataset was used to plot the summarized phase diagram in Fig. 4*B*.

Additional MR Data

Figures S9–S10 show similar angular MR anisotropy for two other samples grown on Element 6 optical-grade diamond substrates. The symmetry relationships of R_{xx}, $R_{xy,\text{asym}}$, and $R_{xy,\text{sym}}$ follow the same trends as those presented for AE-1 in the main text. However, it is notable that the anisotropic symmetry of E-16 does not show any lobed symmetry with respect to current flow direction and instead shows a simple decrease in resistance as the field is rotated OP-to-IP. This suggests that the statistical variation in boron doping across the sample does not play a significant role in the anisotropy. Instead, vicinity to the critical boron concentration might be more relevant to observe these anomalous transport phenomena that arise to the competition between electron correlation and electron-phonon coupling.

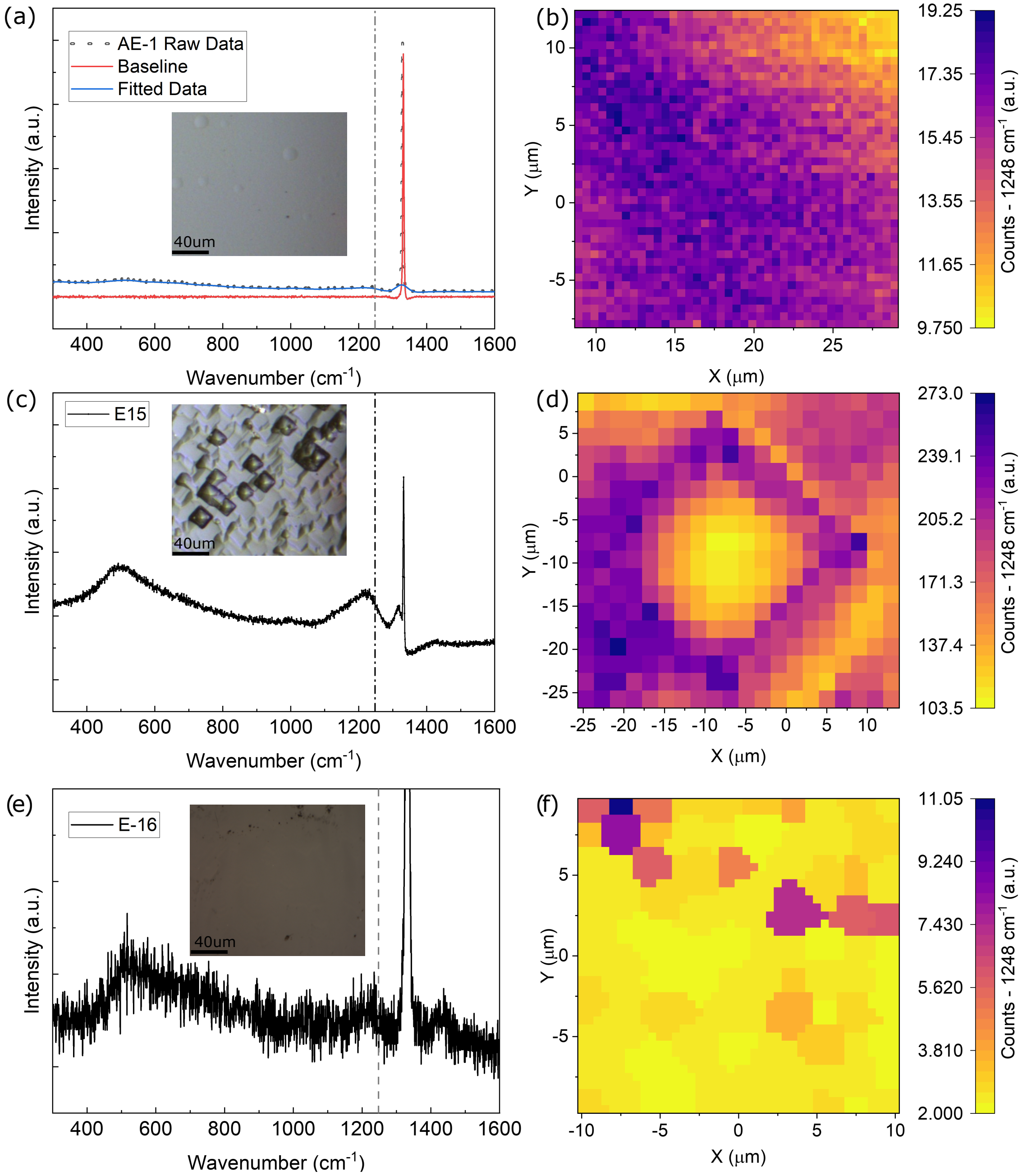

Fig. S1. (a) Baseline subtraction procedure for Raman analysis and the corresponding spectra for sample AE-1. Inset shows the optical image of the sample with a smooth surface (R_q - 2.32 nm) (b) 1248 cm^{-1} peak area heat map for a 20 μm × 20 μm area recorded with a step size of 500 nm on AE-1 (0.5 μm thick sample). No spatial variation in peak intensity is observed. (c) Raw Raman spectrum of sample E-15 that shows complementary magnetoresistance (Fig. S10) anisotropies as compared to AE-1. Inset shows the optical image of the sample with a surface riddled with pits ($R_q > 50$ nm) (d) 1248 cm^{-1} peak area heat map for a 20 μm × 20 μm 'pit' recorded with a step size of 500 nm on sample E-15 (20 μm thick sample grown on an optical-grade substrate). Large spatial variation in peak intensity is observed. (e) Raw Raman spectrum of Sample E-16 shows low boron concentration with non-uniformity and the small peak around 1450 cm^{-1} indicates the formation of amorphous carbon. The magnetoresistance of this sample is less anisotropic than the other two cases. (e) 1248 cm^{-1} peak area heat map for E-16.

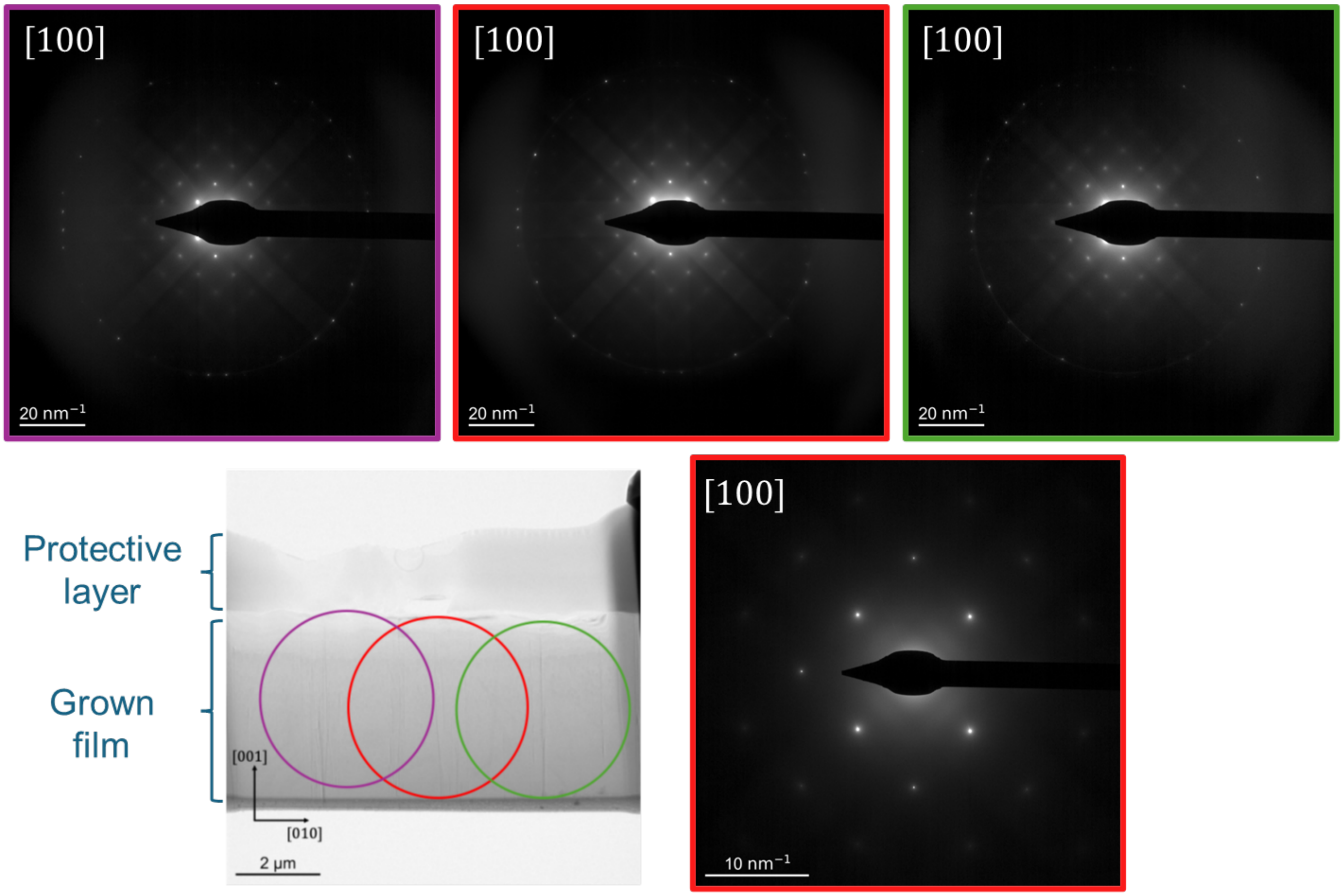

Fig. S2. Selected area electron diffraction (SAED) analysis of a cross section that shows the same (100) orientation across a greater than 9-µm wide region of the boron doped diamond layer (E-14) grown on a (100) optical grade substrate (i.e. homoepitaxial single crystal growth). Top: three SAED patterns from the overlapping regions marked in purple, red, and green in the TEM image below. Bottom: TEM image of the cross section analyzed (with the regions for SAED analysis marked) and a zoomed-in SAED pattern of the area within the red circle.

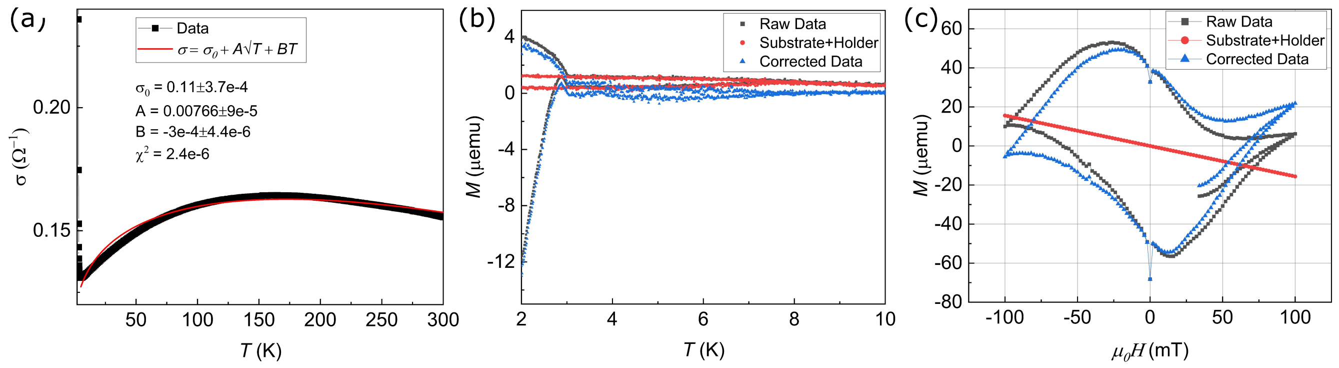

Fig. S3. a. Temperature dependence of conductivity fit to the behavior expected in a disordered 3D metal from quantum corrections dominated by electron-electron interactions. b, c. Correction protocol for M-T, M-H data

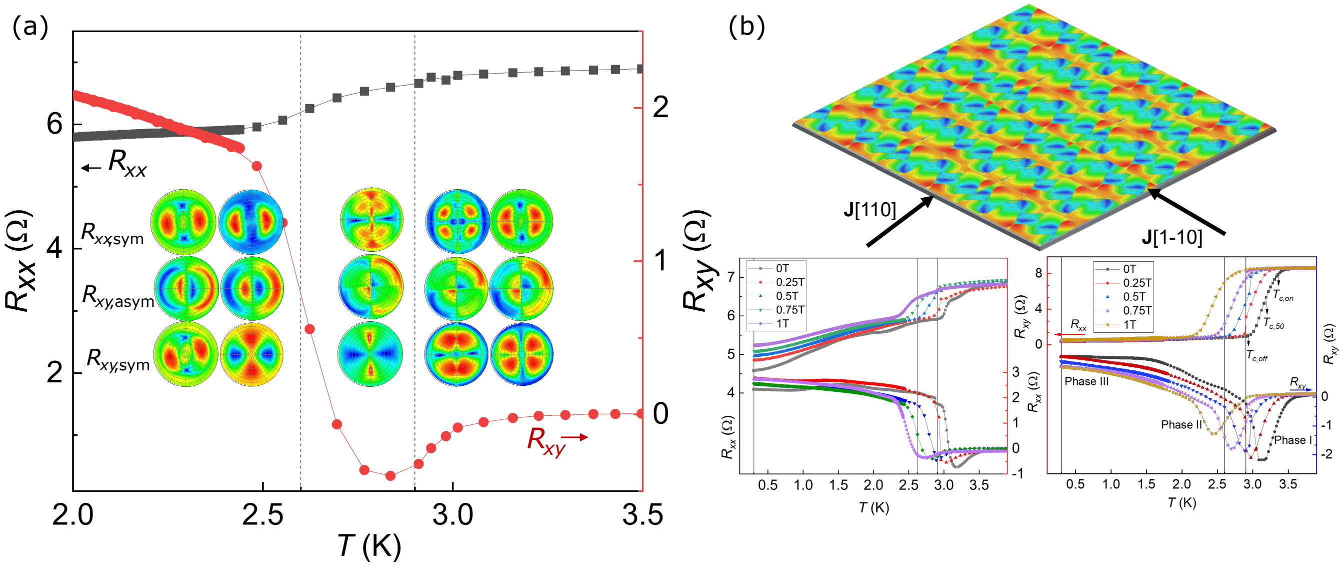

Fig. S4. (a) Polar plots for three phase magnetoresistance anisotropy as observed for ***J*** ∥ (110). (b) Visual representation of ordered bosonic/fermionic puddles based on current direction dependent transport anisotropy. Note that the red regions block the zero resistance paths for ***J*** ∥ (110) direction.

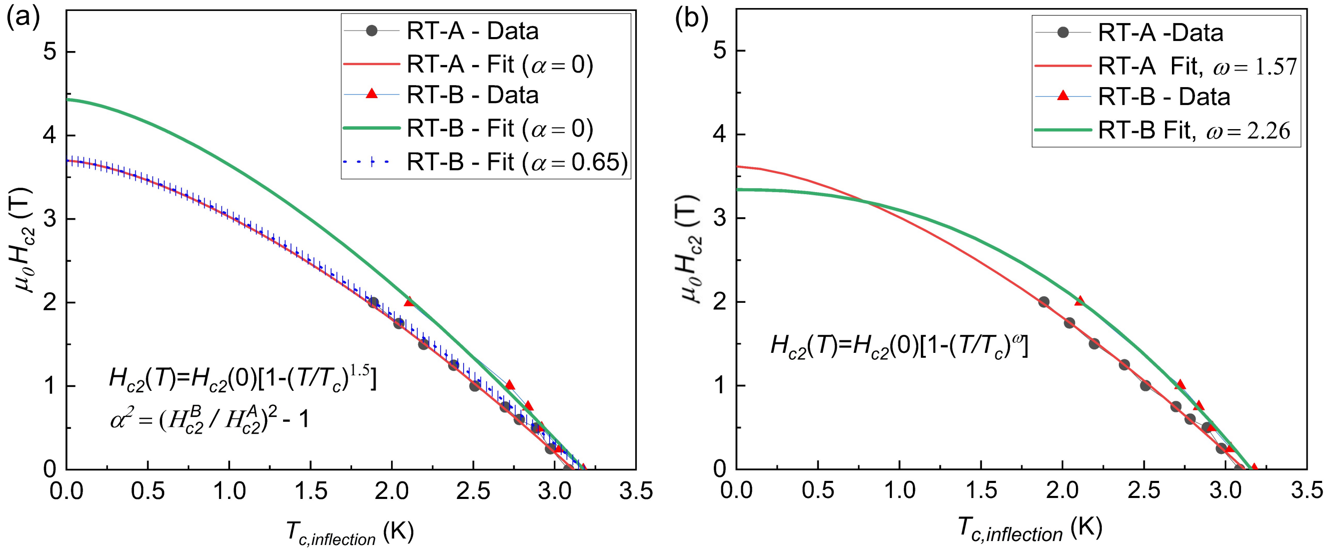

Fig. S5. In both figures RT-A corresponds to ***J*** || (1-10) while RT-B corresponds to ***J*** || (110). **A**. WHH fit considering BCS exponent of 1.5. **B**. WHH fit taking the exponent as a fitting parameter.

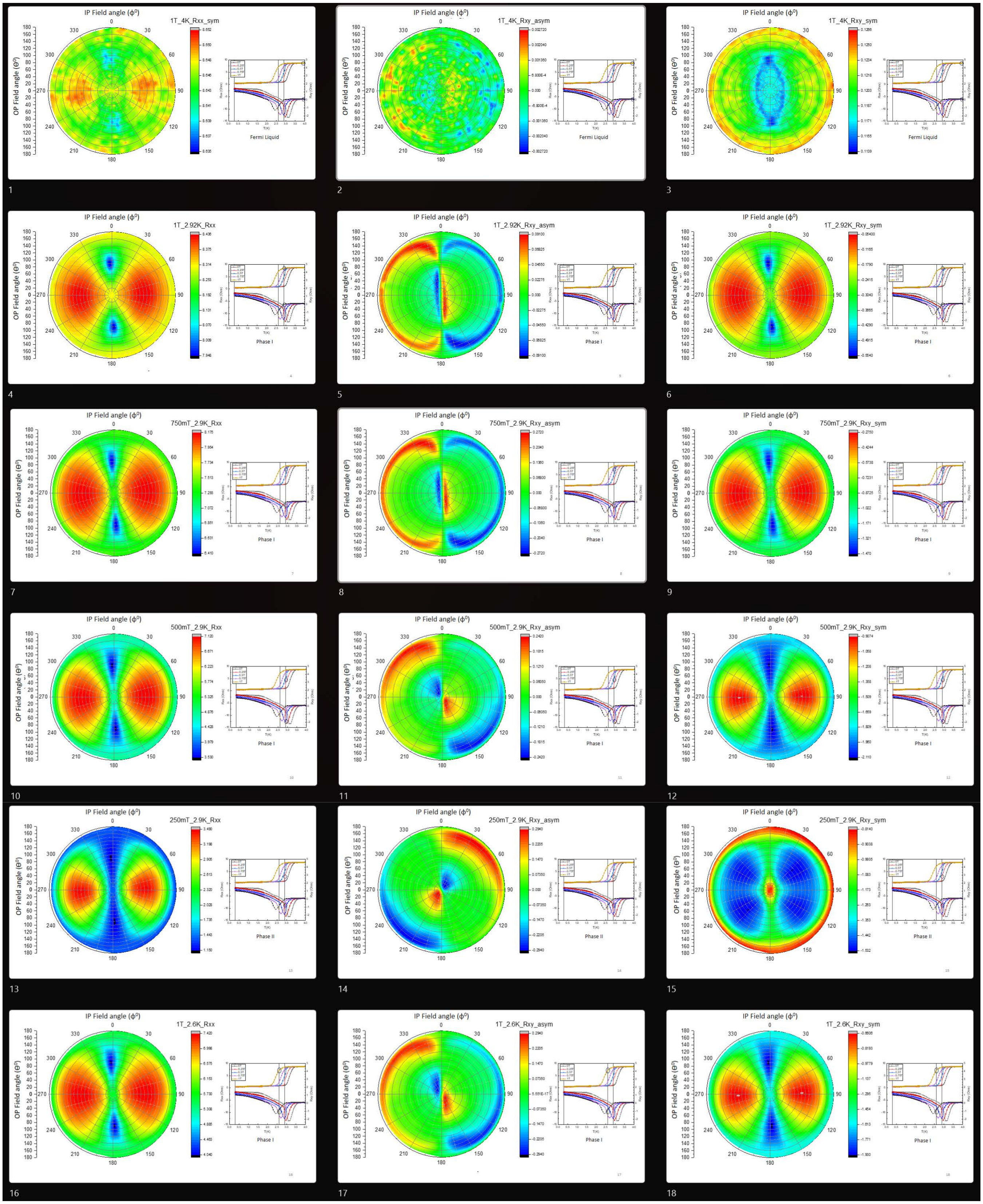
IP Field angle (ϕ⁰)
OP Field angle (θ⁰)
1T_4K_Rxx_sym
1T_4K_Rxy_asym
1T_4K_Rxy_sym
Fermi Liquid
1T_2.92K_Rxx
1T_2.92K_Rxy_asym
1T_2.92K_Rxy_sym
750mT_2.9K_Rxx
750mT_2.9K_Rxy_asym
750mT_2.9K_Rxy_sym
500mT_2.9K_Rxx
500mT_2.9K_Rxy_asym
500mT_2.9K_Rxy_sym
250mT_2.9K_Rxx
250mT_2.9K_Rxy_asym
250mT_2.9K_Rxy_sym
1T_2.6K_Rxx
1T_2.6K_Rxy_asym
1T_2.6K_Rxy_sym
Phase I
Phase II
1
2
3
4
5
6
7
8
9
10
11
12
13
14
15
16
17
18

Fig. S6

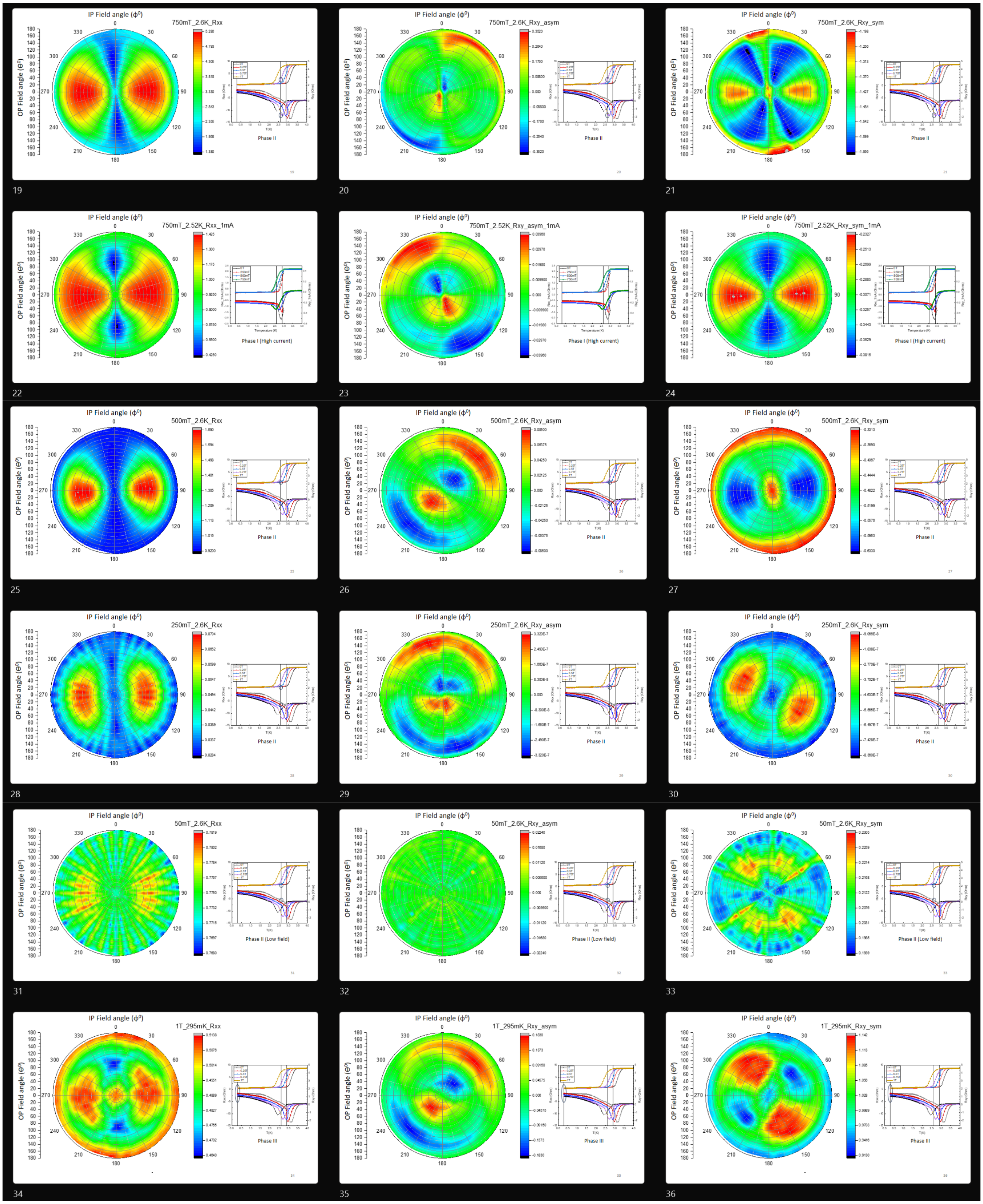
750mT_2.6K_Rxx
750mT_2.6K_Rxy_asym
750mT_2.6K_Rxy_sym
750mT_2.52K_Rxx_1mA
750mT_2.52K_Rxy_asym_1mA
750mT_2.52K_Rxy_sym_1mA
500mT_2.6K_Rxx
500mT_2.6K_Rxy_asym
500mT_2.6K_Rxy_sym
250mT_2.6K_Rxx
250mT_2.6K_Rxy_asym
250mT_2.6K_Rxy_sym
50mT_2.6K_Rxx
50mT_2.6K_Rxy_asym
50mT_2.6K_Rxy_sym
1T_295mK_Rxx
1T_295mK_Rxy_asym
1T_295mK_Rxy_sym
IP Field angle (ϕ⁰)
OP Field angle (θ⁰)
Phase I (High current)
Phase II
Phase II (Low field)
Phase III
19
20
21
22
23
24
25
26
27
28
29
30
31
32
33
34
35
36

Fig. S7

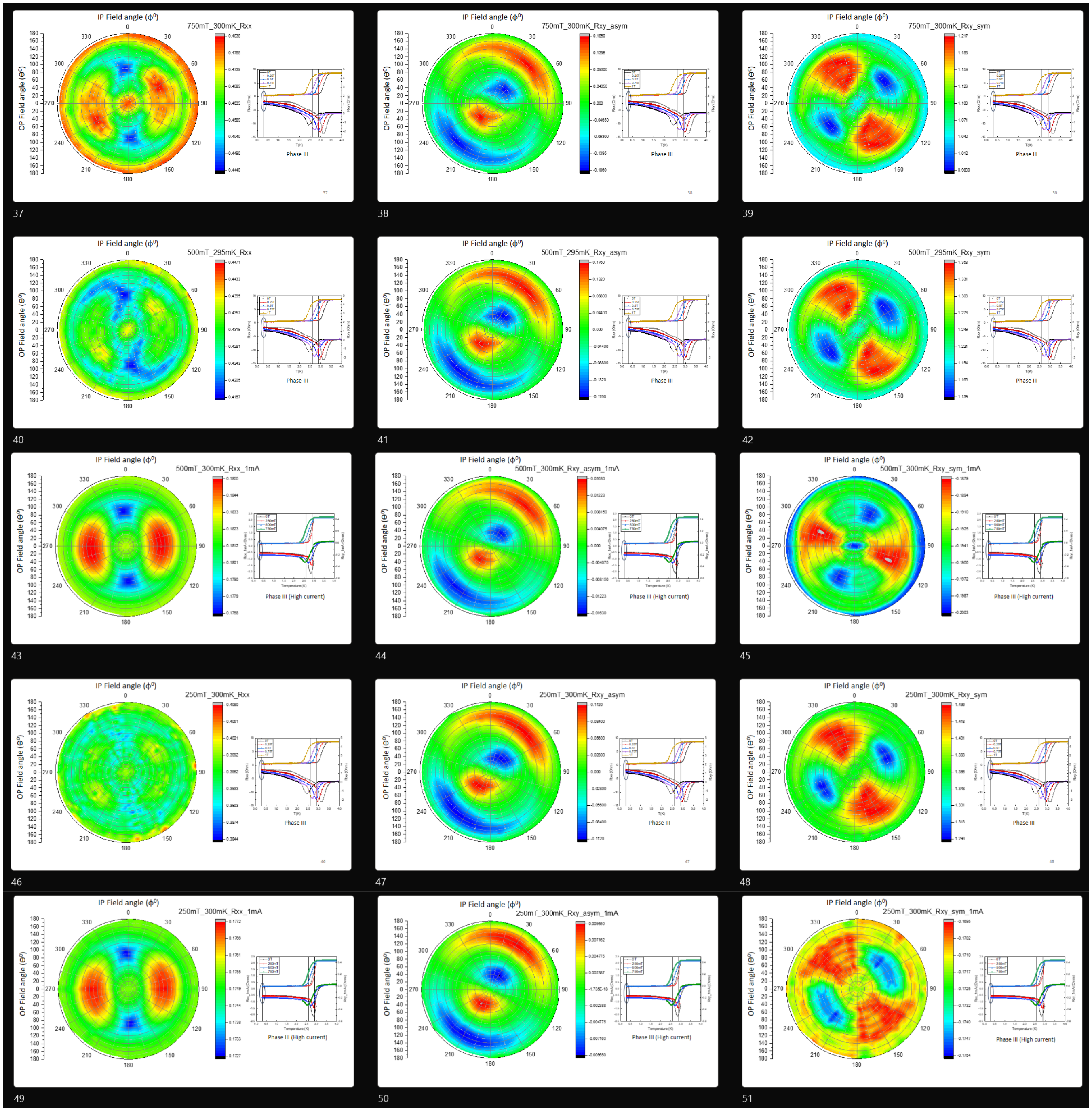
IP Field angle (ϕ⁰)
OP Field angle (θ⁰)
750mT_300mK_Rxx
Phase III
37
750mT_300mK_Rxy_asym
38
750mT_300mK_Rxy_sym
39
500mT_295mK_Rxx
40
500mT_295mK_Rxy_asym
41
500mT_295mK_Rxy_sym
42
500mT_300mK_Rxx_1mA
Phase III (High current)
43
500mT_300mK_Rxy_asym_1mA
44
500mT_300mK_Rxy_sym_1mA
45
250mT_300mK_Rxx
46
250mT_300mK_Rxy_asym
47
250mT_300mK_Rxy_sym
48
250mT_300mK_Rxx_1mA
49
250mT_300mK_Rxy_asym_1mA
50
250mT_300mK_Rxy_sym_1mA
51

Fig. S8

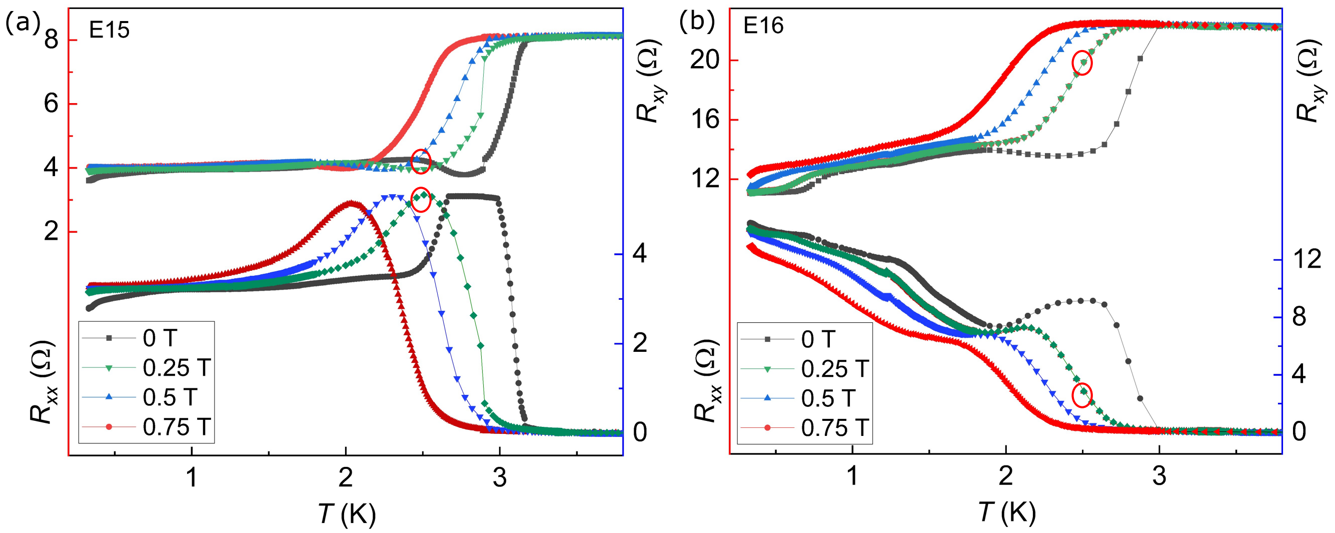

Fig. S9. A (B). Resistance vs temperature plots for sample E-15 (E-16) at different OP magnetic fields. Points circled in red denote the conditions for the MR plots presented in Figure SS10.

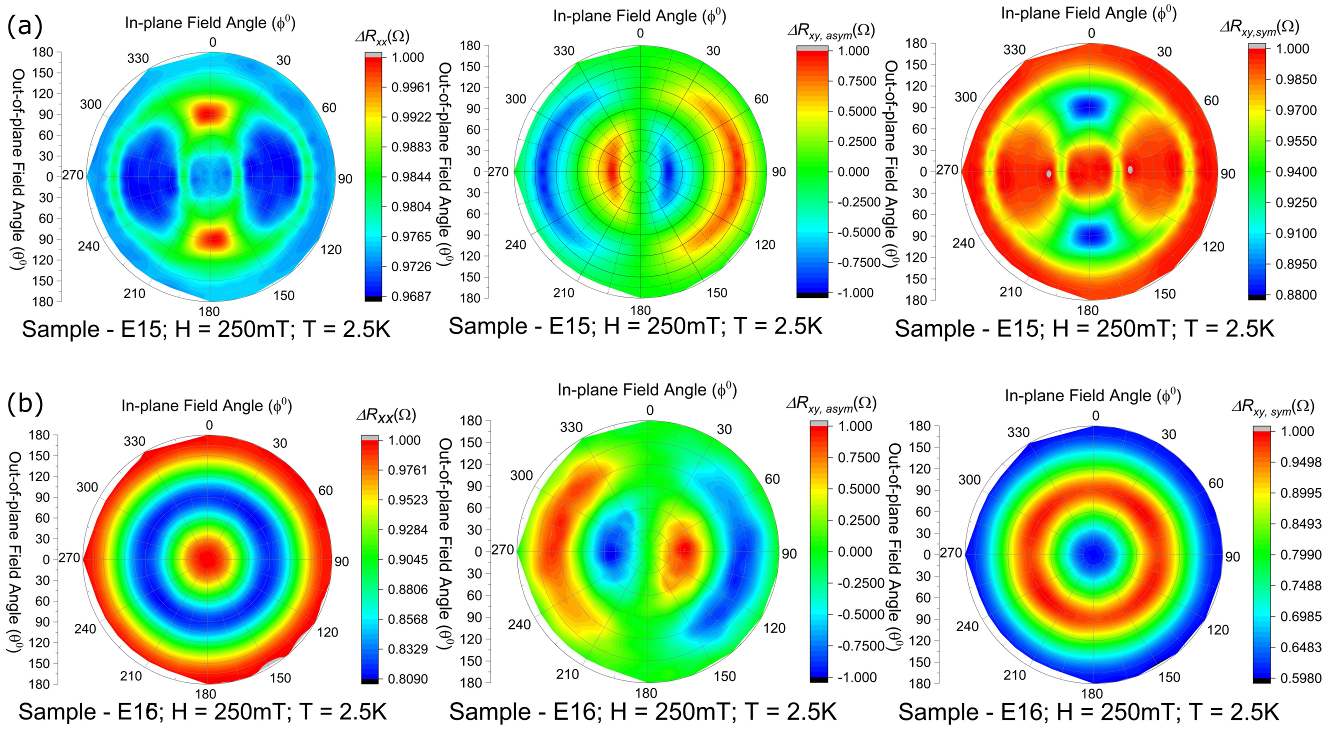

Fig. S10. A (B). Angular MR plots for sample E-15 (E-16) for H = 250 mT, T = 2.5 K. Note that the symmetry of R_{xx} and $R_{xy,\mathrm{sym}}$ plots for both samples is complementary and dR_{xy}/dT and dR_{xx}/dT are approximately 90° relative to each other (Fig. S9) in this phase.

	Peak Position	Height	FWHM	Fano asymmetry
Peak 1	311.77 ± 2.26	40.89 ± 3.46	55.59 ± 6.03	8.12 x 10^{21} ± –
Peak 2	506.15 ± 1.12	40.07 ± 3.36	57.55 ± 4.21	1.81 x 10^{7} ± –
Peak 3	666.97 ± 14.46	102.75 ± 6.53	314.66 ± 13.65	-2.54± 0.28
Peak 4	657.35 ± 10.86	8.43 ± 2.74	50.76 ± 15.73	-50.87 ± 467.40
Peak 5	1015.17 ± 3.16	11.36 ± 0.58	30.65 ± 3.15	-9.67 ± 5.20
Peak 6	1248.02 ± 0.52	40.55 ± 0.67	45.12 ± 0.95	-1.33 ± 0.012
Peak 7	1330.17 ± 0.26	121.96 ± 0.73	17.81 ± 0.23	-7.45677 ± 0.42

Table S1. Peak Parameters for BWF Raman Fit

**Parameter** (2 K)	Mott Metal	Phase I	Phase II	Phase III
Magnetic field range	$\mu_0 H > 3\ \mathrm{T}$	$2.2\ \mathrm{T} < H < 3\ \mathrm{T}$	$1\ \mathrm{T} < \mu_) H < 2.2\ \mathrm{T}$	$0 < \mu_0 H < 1\ \mathrm{T}$
R/H (Ohm/T)	0.00285	0.017	0.03078	N/A
Carrier type	Hole	Electron	Hole	Cooper pair
Carrier density (cm^{-3})	4.38 $\times 10^{21}$	7.34 $\times 10^{20}$	4.05 $\times 10^{20}$	-
Mobility (cm^2/V·s)	11.4	6.8	12.31	-
Mean free path (l_e)(nm)	0.473	2.82	5.11	$\zeta \sim$ 9
$k_F = (3\pi^2 n)^{1/3}\ (\mathrm{m}^{-1})$	5.06×10^9	2.77×10^9	2.29×10^9	N/A
$k_F l_e$	2.39	7.81	11.7	N/A

Table S2. Summary of carrier properties extracted from Fig. 3(b)

**Sample**	**Gas Flow** $H_2/CH_4/BCl_3 - Ar$**(1000ppm)(sccm)**	**Temp (℃)**	**Time min**	**Thickness (μm)**	**AFM Ra nm**	$T_{c,\text{onset}}$ **K**
E14	190/10/50	1050	60	~ 25	50	3 K
E15	190/10/70	1170	40	~ 0.5	NA	3.2 K
E16	190/5/50	1050	50	~ 2	24	3 K
E17	190/5/70	1400	35	~ 2	4.85	5.5 K
E18	95/2.5/75	1100	45	~1	2.3	2.8 K
E19*	95/5/50	1150	15	NA	20	2.8 K
E20**	95/5/75	1100	15	~16	280	3.2K
E21v2*	95/2.5/50	1050	60	~ 4	40	3.2 K
E22*	95/5/200	NA	20	~ 7	52	2 K
AE1	95/5/75	1230	25	~ 0.5	2.3	2.8 K
AE2	95/2.5/125	1150	60	~ 12	0.8	Insulating

Table S3. Summary of HBDD samples grown. All samples in the Exx category were grown on optical-grade (100) diamond substrates. The two samples in the AExx category were grown on electronic-grade (100) diamond substrates.

References

1. V Mortet, et al., New perspectives for heavily boron-doped diamond Raman spectrum analysis. *Carbon* **168**, 319 (2020).
2. R Milligan, T Rosenbaum, R Bhatt, G Thomas, Chapter 3 - a review of the metal–insulator transition in doped semiconductors in *Electron–Electron Interactions in Disordered Systems*, Modern Problems in Condensed Matter Sciences, eds. A Efros, M Pollak. (Elsevier) Vol. 10, pp. 231–286 (1985).
3. J Bousquet, et al., Phase diagram of boron-doped diamond revisited by thickness-dependent transport studies. *Phys. Rev. B* **95**, 161301 (2017).
4. G Klemencic, et al., Phase slips and metastability in granular boron-doped nanocrystalline diamond microbridges. *Carbon* **175**, 43 (2021).
5. G Zhang, et al., Bosonic Confinement and Coherence in Disordered Nanodiamond Arrays. *ACS Nano* **11**, 11746 (2017).
6. G Zhang, et al., Bosonic Anomalies in Boron-Doped Polycrystalline Diamond. *Phys. Rev. Appl.* **6**, 064011 (2016).
7. G Zhang, et al., Global and local superconductivity in boron-doped granular diamond. *Adv. Mater.* **26**, 2034 (2014).
8. A Segal, M Karpovski, A Gerber, Inhomogeneity and transverse voltage in superconductors. *Phys. Rev. B* **83**, 094531 (2011).
9. Y Cao, et al., Nematicity and competing orders in superconducting magic-angle graphene. *Science* **372**, 264 (2021).
10. T Kageura, et al., Superconductivity in nano- and micro-patterned high quality single crystalline boron-doped diamond films. *Diam. Relat. Mater.* **90**, 181–187 (2018).
